# Ultrahigh frequencies of peripherally matured LGI1- and CASPR2-reactive B cells characterize the cerebrospinal fluid in autoimmune encephalitis

**DOI:** 10.1073/pnas.2311049121

**Published:** 2024-02-06

**Authors:** Jakob Theorell, Ruby Harrison, Robyn Williams, Matthew I. J. Raybould, Meng Zhao, Hannah Fox, Andrew Fower, Georgina Miller, Zoe Wu, Eleanor Browne, Victor Mgbachi, Bo Sun, Rohini Mopuri, Ying Li, Patrick Waters, Charlotte M. Deane, Adam Handel, Mateusz Makuch, Sarosh R. Irani

**Affiliations:** ^a^Oxford Autoimmune Neurology Group, Nuffield Department of Clinical Neurosciences, University of Oxford, Oxford OX3 9DU, United Kingdom; ^b^Department of Medicine Huddinge, Karolinska Institutet, Stockholm 17177, Sweden; ^c^Department of Neurology, Karolinska University Hospital, Stockholm 17176, Sweden; ^d^Department of Neurology, John Radcliffe Hospital, Oxford University Hospitals, Oxford OX3 9DU, United Kingdom; ^e^Department of Statistics, Oxford Protein Informatics Group, University of Oxford, Oxford OX1 3LB, United Kingdom; ^f^Department of Quantitative Health Sciences, Mayo Clinic, Jacksonville, FL 32224; ^g^Departments of Neurology and Neuroscience, Mayo Clinic, Jacksonville, FL 32224

**Keywords:** autoantibody, encephalitis, cerebrospinal fluid, plasmablast, autoantigen

## Abstract

Intrathecal synthesis of central nervous system (CNS)-reactive autoantibodies is observed across patients with autoimmune encephalitis (AE), who show multiple residual neurobehavioral deficits and relapses despite immunotherapies. We leveraged two common forms of AE, mediated by leucine-rich glioma inactivated-1 (LGI1) and contactin-associated protein-like 2 (CASPR2) antibodies, as human models to comprehensively reconstruct and profile cerebrospinal fluid (CSF) B cell receptor (BCR) characteristics. We hypothesized that the resultant observations would both inform the observed therapeutic gap and determine the contribution of intrathecal maturation to pathogenic B cell lineages. From the CSF of three patients, 381 cognate-paired IgG BCRs were isolated by cell sorting and scRNA-seq, and 166 expressed as monoclonal antibodies (mAbs). Sixty-two percent of mAbs from singleton BCRs reacted with either LGI1 or CASPR2 and, strikingly, this rose to 100% of cells in clonal groups with ≥4 members. These autoantigen-reactivities were more concentrated within antibody-secreting cells (ASCs) versus B cells (*P* < 0.0001), and both these cell types were more differentiated than LGI1- and CASPR2-unreactive counterparts. Despite greater differentiation, autoantigen-reactive cells had acquired few mutations intrathecally and showed minimal variation in autoantigen affinities within clonal expansions. Also, limited CSF T cell receptor clonality was observed. In contrast, a comparison of germline-encoded BCRs versus the founder intrathecal clone revealed marked gains in both affinity and mutational distances (*P* = 0.004 and *P* < 0.0001, respectively). Taken together, in patients with LGI1 and CASPR2 antibody encephalitis, our results identify CSF as a compartment with a remarkably high frequency of clonally expanded autoantigen-reactive ASCs whose BCR maturity appears dominantly acquired outside the CNS.

## Significance

In CNS autoantibody-mediated diseases, it is unknown where B cells mature or how autoantibodies access the CNS. To examine CSF (cerebrospinal fluid) BCR (B cell receptor) maturation, we studied LGI1- and CASPR2-antibody encephalitis as model diseases. Systematic reconstruction of CSF BCR repertoires highlighted a dominant representation of antibody-secreting cells (ASCs) with marked enrichments for LGI1 or CASPR2 reactivities, universally in larger clonal expansions. Clonally-expanded BCRs had undergone limited intrathecal affinity maturation or mutational activity, alongside low T cell clonality. In contrast, significant LGI1 and CASPR2 affinity was acquired between the germline and intrathecal BCRs. Hence, ultrahigh enrichments of CSF autoantigen-specific ASCs are likely to contribute pathogenic autoantibodies, yet these cells appear to dominantly diversify and mature outside the CNS.

A recent explosion in the number of central nervous system (CNS) autoantibody-mediated diseases has made understanding their underlying immunopathogenesis an increasingly important and transferable endeavor, with the potential to identify both biologically fundamental and druggable processes (1–4). Consistent with their direct pathogenic potential, these autoantibodies bind the extracellular domains of native human CNS proteins, and their passive transfer to experimental animals (5–7) reproduces key neuropsychiatric features observed in the patients (1, 2, 4).

The commonest form of autoimmune encephalitis (AE) is mediated by leucine-rich glioma inactivated-1 (LGI1) antibodies, and typically affects patients over 60 y of age with unremarkable routine cerebrospinal fluid (CSF) investigations (8–11). These characteristics, alongside several other clinical features, are shared by patients with contactin-associated protein-like 2 (CASPR2) antibody encephalitis (8, 12). After treatment with immunotherapies, up to 40% of patients with both LGI1 and CASPR2 antibody encephalitis experience relapses (11, 13) and residual neurocognitive impairments, which affect quality of life and lead to disability (14, 15).

To help address these unmet medical needs, we sought to understand a critical biological step in the pathogenesis of LGI1 and CASPR2 antibody encephalitis: namely how the pathogenic antibodies access and mature within the CSF. This is particularly relevant in LGI1 antibody encephalitis, as intrathecal LGI1 antibody synthesis associates with a poor prognosis (16). Yet, it remains uncertain whether the dominant contribution to CSF antibodies comes from serum autoantibodies penetrating the CNS or from the secretion of autoantibodies by autoantigen-reactive antibody-secreting cells (ASCs) residing within the CSF. The latter mechanism is supported by autoantigen-specific B cell receptors (BCRs) observed at frequencies of between 5% and 55% from unselected B cells and ASCs within the CSF of patients with AE, including LGI1 antibody encephalitis (17–20), coupled with the paucity of these cells in healthy control CSF (10, 21).

In AE patients, it is not understood whether intrathecal B cells and ASCs divide or affinity mature in the CNS. Meningeal tertiary lymphoid structures (TLS) represent a proposed site of CNS B cell maturation in multiple sclerosis, another CNS autoimmune disease in which pathogenic B cells and autoantibodies are reported (21–23). In multiple sclerosis, and in several tumors, these TLS may act as ectopic germinal centers (GCs), classical sites where BCR somatic hypermutation, affinity maturation, and immunoglobulin class-switch recombination occur, typically with T cell help (23–26). In AE, CNS-resident TLS within the CNS would represent a compartmentalized site of autoantibody maturation which is relatively resistant to therapeutics. Moreover, specific profiling of the autoantigen-reactive cells in CSF presents a “precision medicine” opportunity to selectively target this population.

Motivated by these biological observations and their potential for clinical translation, we characterized and enumerated autoantigen-specificities and dynamics of 166 B-lineage cells in the CSF of patients with LGI1 and CASPR2 antibody encephalitis. We asked whether gene expression profiles might permit highly specific therapeutic targeting of the autoantigen-reactive populations and studied germline BCRs, whole transcriptomes, and T cell receptor (TCR) clonalities to evaluate the relative contributions of peripheral versus CNS processes to CSF BCR maturation and diversification.

## Results

### Patient Characteristics and Lymphocyte Workflow.

**Fig. 1.**
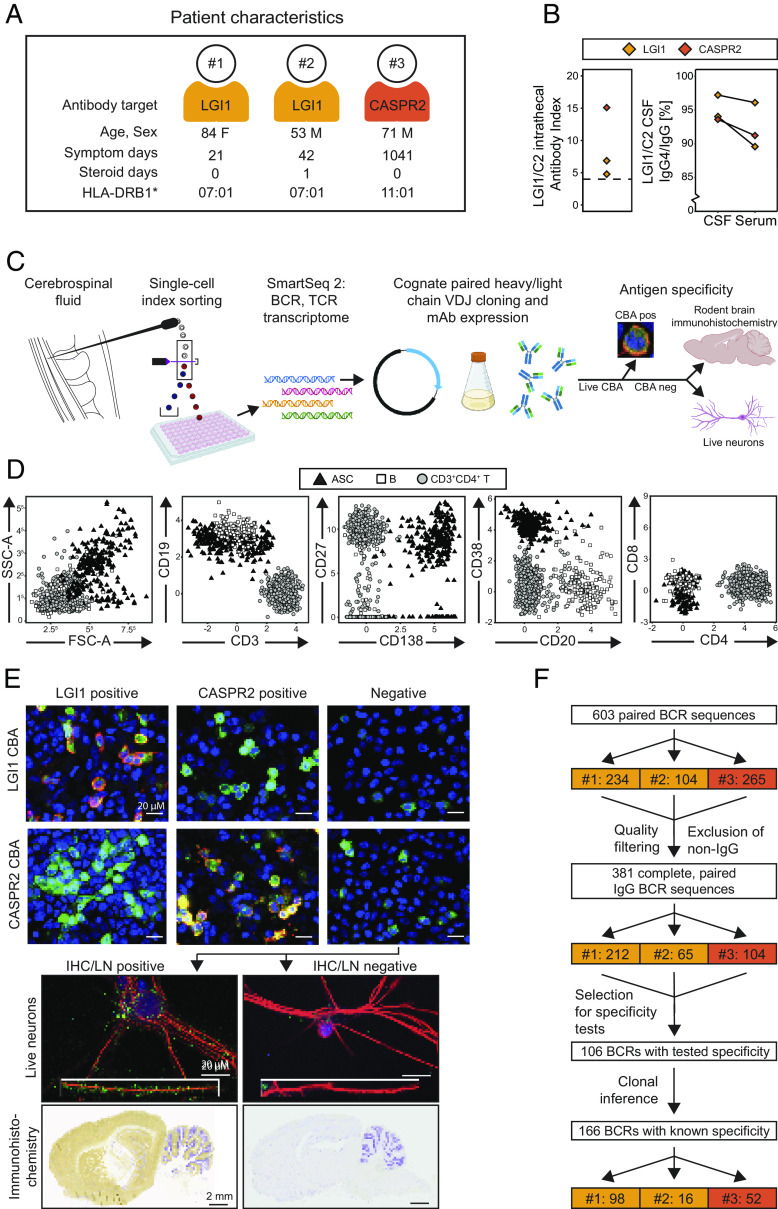
CSF lymphocyte, polyclonal, and mAb findings from patients with LGI1 and CASPR2 antibody encephalitis. (*A*) CSF from patients with acute LGI1 (n = 2; gold) and CASPR2 antibody (n = 1; orange) encephalitis was sampled. Only one patient received any immunotherapy prior to sampling, corticosteroids for 1 d. Demographics, symptom duration, and HLA-DRB1 genotypes are shown. and further clinical details presented in *SI Appendix*, Table S1. (*B*) LGI1 (gold) and CASPR2-IgG (orange) intrathecal antibody indexes (*Left*) were calculated as CSF:serum autoantibody levels normalized to the total IgG CSF:serum ratio, with a cutoff (>4) representing intrathecal synthesis. (*Right*) Flow cytometry determined LGI1 or CASPR2 IgG4:IgG ratios in CSF and serum (13). (*C*) Workflow began with CSF collection, and, within 1 h, the fresh CSF pellet underwent indexed fluorescence-activated cell sorting of single cells followed by plate-based scRNA-seq with Smart-seq2, bioinformatic identification of cognate-paired heavy and light chain IgG BCRs, followed by cloning and mAb expression. mAbs were tested for antigenic specificity, initially by live cell-based assays (CBA) and, if negative, using rodent brain immunohistochemistry and live neurons (representative examples below). (*D*) After exclusion of CD14^+^ and dead cells, ASCs (black triangles) were defined as either CD3^−^CD138^+^ or CD3^−^CD19^+^CD20^−^CD38^+^ with comparatively high side and forward scatter (panel 1). B cells (white squares) were CD3^−^CD19^+^ non-ASCs, and CD4^+^ helper T cells were CD3^+^CD4^+^CD138^-^ (gray circles). (*E*, *Upper*) Cloned, expressed mAbs were tested for binding to HEK293T cells surface-expressing LGI1 (*Top* row) or CASPR2 (*Bottom* row) by CBAs. Representative results of LGI1 antibody positivity (*Left* column) and CASPR2-antibody positivity (*Middle* column) are shown. DAPI staining in blue, LGI1 or CASPR2 tagged with EGFP (green), and AF594 goat anti-human IgG in red. (Scale bar 20 µM.) Next, mAbs with no binding to either LGI1 or CASPR2 (*Right* column, “Negative”) were tested (*Lower*) for binding to the surface of live hippocampal neurons (LN; *Top* row; DAPI staining in blue, red staining for MAP2 to reveal neuronal structure and green represents bound human IgG; *Inset* 125% magnification of a dendrite; scale bar 20 µM) and on rodent immunohistochemistry (IHC; *Bottom* row; bound human IgG developed with 3,3′-Diaminobenzidine; brown) counterstained with Cresyl violet (purple; scale bar 2 mm.) Positive and negative examples of both LN and IHC in the *Left* and *Right* columns, respectively. (*F*) Workflow after scRNA-Seq, showing that 603 paired BCR sequences (n = 234, 104, and 265 for patients #1, 2, and 3, respectively) were filtered to 381 complete and paired BCR sequences, of which 106 were expressed and tested, with clonal inference permitting reporting of 166 BCRs with known antigenic specificities (extended flowchart presented in *SI Appendix*, Fig. S1*B*).

CSF was collected from three patients with either LGI1 (n = 2) or CASPR2 (n = 1) antibody encephalitis at their disease presentation, 21 to 1,041 d after the onset of frequent focal seizures and memory disturbances typical of their disorders (8) (Fig. 1*A* and *SI Appendix*, Table S1). All had unremarkable CSF leucocyte counts, by routine clinical assessments (Table S1). Two were untreated, and one had received corticosteroids for just 1 d, meaning that medications were unlikely to interfere with the underlying immunology. Flow cytometric analyses of CSF IgGs (13) consistently identified a raised LGI1 or CASPR2 antibody index and 94 to 97% of the CSF LGI1 and CASPR2 antibodies were of the IgG4 subclass, greater proportions than observed in serum (Fig. 1*B*). These metrics highlighted patient CSF as a distinctive compartment, with preferential intrathecal synthesis of the autoantigen-specific antibodies, and strongly indicated the presence of CSF autoantigen-reactive B-lineage cells.

To enumerate and characterize these cells individually, we designed a single-cell workflow which evaluated 1) lymphocyte surface phenotypes; 2) their whole transcriptomes and antigen receptor (TCR and BCR) sequences; and 3) their BCR specificities, by assembling the cognate heavy–light chain pairs as monoclonal antibodies (mAbs; Fig. 1 *C*–*E*). All mAbs were first tested against the extracellular domain of HEK293T-expressed LGI1 or CASPR2, using live cell-based assays (8, 14). If unreactive to both, mAb binding was assessed against broader CNS antigenic reactivities expressed on the surface of live rodent hippocampal neurons and on fixed rodent brain sections (Fig. 1*E*) (7, 14).

To maximize recovery of B-lineage cells from fresh CSF, particularly ASCs which often show high granularity and size (Fig. 1*D*) and are most likely to contribute to the observed intrathecal antibody synthesis, a strategy was designed to index sort CD45^+^ and/or CD138^+^ live CD14^−^ single cells and to enrich for CD19^+^ cells and large CD138^+^ leukocytes (Fig. 1*D* and *SI Appendix*, Fig. S1*A*). Data from additional surface markers (CD20, CD38, CD27, IgD, CD3, CD4, and CD8) were simultaneously collected alongside forward and side scatter characteristics. Collectively, these were used to define ASCs as CD3^−^CD138^+^ or CD3^−^CD19^+^CD20^−^CD38^+^ cells (typically side scatter area^hi^, forward scatter area^hi^), B cells as CD3^−^CD19^+^ non-ASCs, and CD4^+^ T cells as CD3^+^CD4^+^CD138^-^, within the CD45^+^CD14^−^ live population.

### B-Lineage Clonal Expansions Closely Associate with LGI1 or CASPR2 Reactivities.

Next, to deeply characterize the individual lymphocytes isolated by index cell sorting, plate-based Smart-seq2 scRNA-seq was undertaken. This generated whole transcriptome libraries with a median of 949,269 total and 3,914 unique expressed genes per cell, higher than observed with droplet-based methodologies (27). After computational filtering, a total of 381 cells were identified with full-length cognate-paired heavy and light IgG chains, corresponding to single functional BCRs (Fig. 1*F* and *SI Appendix*, Fig. S1*B*).

**Fig. 2.**
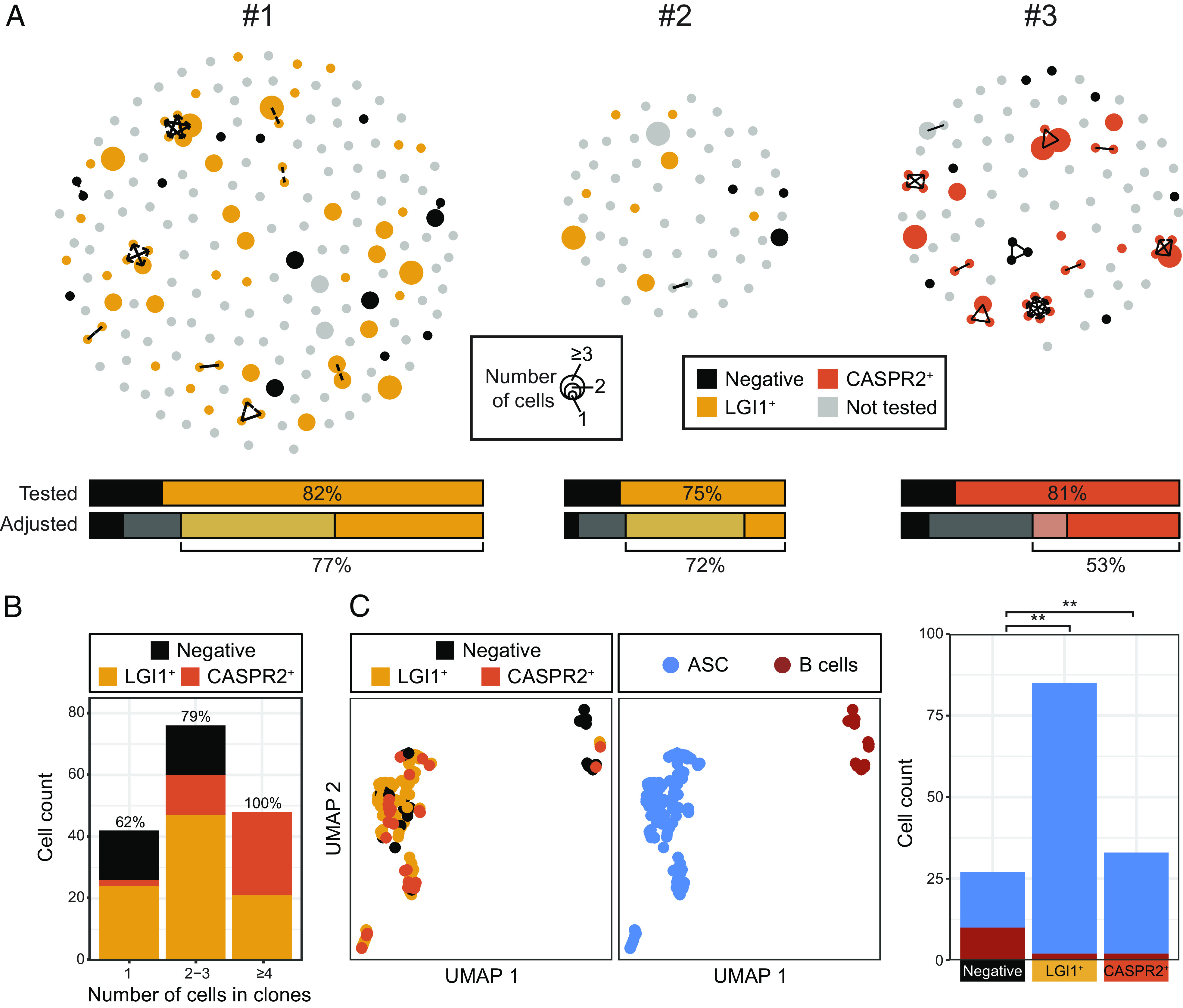
LGI1- and CASPR2-reactive cells are predominantly ASCs and highly enriched in larger clonal groups. (*A*) Individualized BCR data from the three patients showing clustering of clonally related and singleton immunoglobulin heavy chain variable regions from a total of 381 cognate-paired complete BCR sequences (212 from patient #1 with LGI1 antibody encephalitis; 65 from patient #2 with LGI1 antibody encephalitis; 104 from patient #3 with CASPR2 antibody encephalitis). Each dot represents one heavy chain, its size represents the number of times that heavy chain sequence was observed and lines connect clonally related sequences [shared heavy chain V-gene and J-gene, with identical junction length and CDRH3 junction Hamming distance <6, using Immcantation clonal definitions (28)]. A selection of 106 clonal and singleton BCRs were expressed as mAbs and depicted as LGI1 reactive (gold) or CASPR2 reactive (orange) or unreactive (black). Those which were not tested are gray. As clonal members consistently shared antigen reactivities (*SI Appendix*, Fig. S1*B*), data from 166 mAbs are presented. (*B*) Percentage of LGI1- and CASPR2-reactive cells from singletons (62%), clones with 2 to 3 members (79%) or with ≥4 members, showing enrichment to 100% specificity. (*C*, *Left*) UMAP analyses identified LGI1- and CASPR2-specific BCRs are predominantly from ASCs (n = 114 of 131, blue) and cluster separately to B cells (n = 4 of 14; red) and (*Right*) are a higher proportion of ASCs (Fisher’s exact test; *P* = 0.002 for LGI1, *P* = 0.001 for CASPR2 and combined *P* < 0.001).

BCR clustering revealed multiple clonal expansions across the three patients, most marked in patients #1 and #3 (Fig. 2*A*). Initially, as a proof of concept, reactivities of 32 BCRs were studied from nine of these clones, each with 2 to 5 tested members. When expressed as mAbs, blinded assessments revealed that all intraclonal BCRs shared the same reactivities: 29 reacted with LGI1 and three (one clone) bound rodent brain sections, but not LGI1 or CASPR2 (*SI Appendix*, Fig. S1 *B*–*D*). Hence, as expected, members of individual clones showed consistent autoantigen-reactivities.

This allowed us to examine one of our major hypotheses: Clonally expanded populations more frequently harbored autoantigen-reactive BCRs, reflecting their preferential division upon sensing their cognate CNS autoantigens in CSF. To test this concept, we expressed one mAb from each of 64 clonal groups, in addition to 42 randomly selected BCRs from nonclonal cells (“singletons”). Given the observed absolute consistency of intraclonal reactivities (*SI Appendix*, Fig. S1 *C* and *D*), this was extrapolated to represent 166 BCRs in total (Fig. 1*F*).

Strikingly, of these 166 cognate-paired mAbs, 75 to 82% were reactive to either LGI1 (patients #1 and #2) or CASPR2 (patient #3) (Fig. 2*A*, “Tested” upper bars). None bound both LGI1 and CASPR2, and none bound to HEK293T cells expressing another known neurological autoantigen, aquaporin-4 (*SI Appendix*, Fig. S1*D*). This ultrahigh frequency of autoantigen specificity was further enriched in increasingly expanded clones: rising from 62% in singletons, to 79% in clones with 2 to 3 members and, in clones with ≥4 members (range 4 to 8), remarkably, 100% of the cells were either LGI1 or CASPR2 reactive (*P* < 0.0001; Fisher’s exact test; Fig. 2*B*). When adjusting for these frequencies in untested singleton BCRs, the rates of LGI1 or CASPR2 specificity were 53 to 77% (Fig. 2*A*, “adjusted” lower bars). Using immunohistochemistry and live neuron staining, CNS reactivities were detected in only 0 to 12.5% of all B-lineage cells in the two LGI1 antibody encephalitis patients (*SI Appendix*, Fig. S2 *A* and *B*).

Linking these BCR specificities with their FACS (fluorescence-activated cell sorting)-determined cell surface phenotypes, highlighted that the cells with LGI1 or CASPR2 reactivities were greatly enriched within the ASC population, accounting for 114/131 (87%) of all ASCs. By contrast, both LGI1 and CASPR2 reactivities were less abundant within B cell populations (4/14; 29%; Fisher’s exact test *P* = 0.002 (for LGI1) and *P* = 0.001 (for CASPR2); Fig. 2*C* and *SI Appendix*, Fig. S2 *C* and *D*). Taken together, LGI1 and CASPR2 reactivities were strikingly enriched in clonally expanded ASC-dominated CSF populations.

### Transcriptional Differences between Autoantigen-Reactive Versus -Unreactive B Cells and ASCs.

**Fig. 3.**
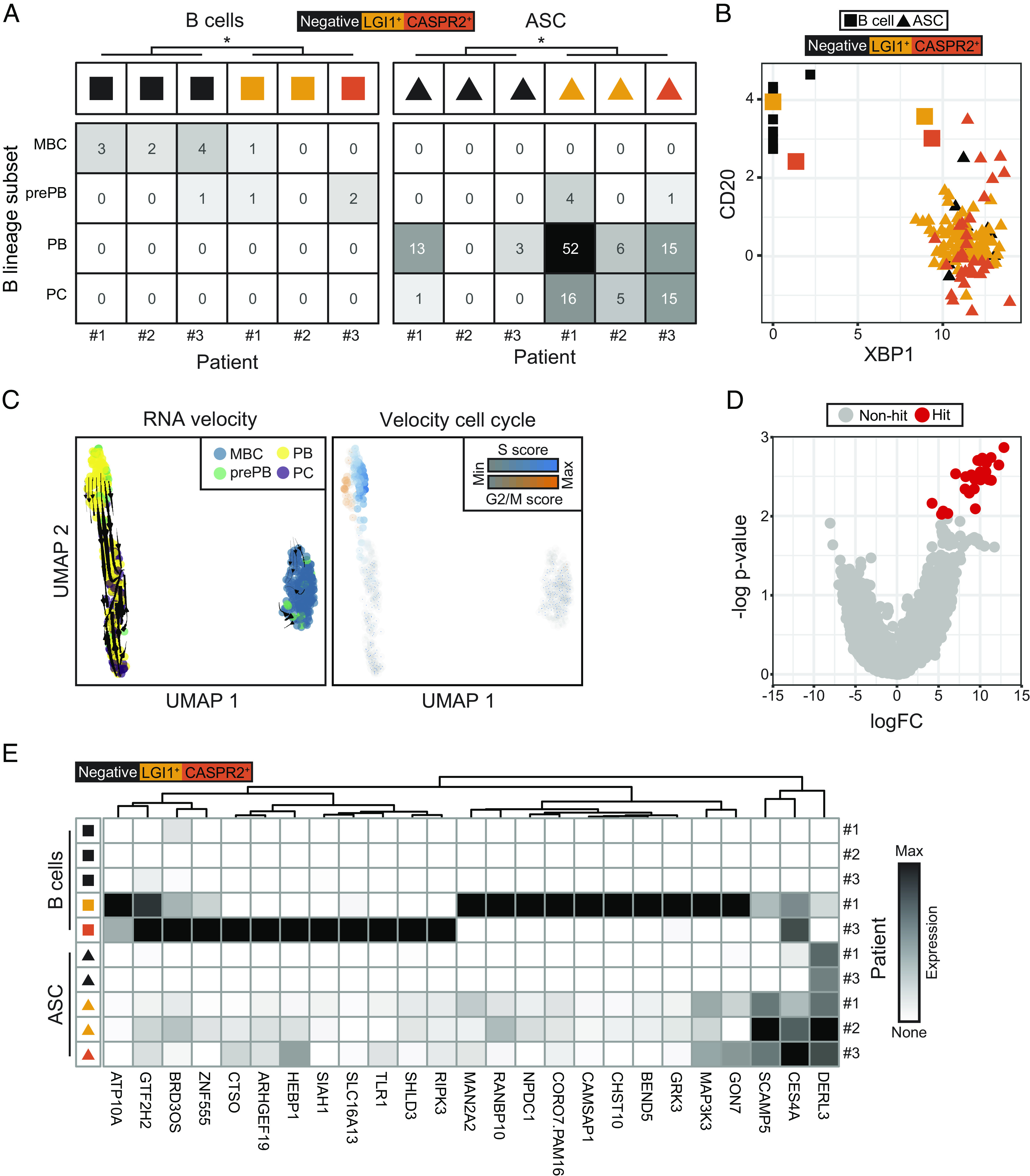
LGI1- and CASPR2-reactive cells are more differentiated than their nonreactive counterparts. (*A*) LGI1- and CASPR2-reactive B cells (colored squares, n = 4) and ASCs (colored triangles, n = 114) demonstrate more differentiated gene expression profiles than their LGI1- and CASPR2-unreactive counterparts (black squares and triangles, n = 10 and n = 17, respectively; Fisher’s exact test for pooled B cells *P* = 0.04 and ASCs *P* = 0.02). Cell population definitions using SingleR, with bulk reference data for gene expression profiles of memory B cell (MBC), prePB (preplasmablast), PB (plasmablast), and (PC) plasma cell populations from Kassambara et al. (29). (*B*) Three of four LGI1- or CASPR2-reactive CD20^+^ B cells demonstrate increased XBP1 transcript expression, compared to one of ten nonreactive B cells (Fisher exact test, *P* = 0.04). (*C*) RNA velocity trajectories (*Left*) show separation of memory B cells (blue, MBC) versus ASCs with consistent directionality, within ASCs, from preplasmablasts (green, prePB) and plasmablasts (yellow, PBs) toward and plasma cells (purple, PCs). (*Right*) Velocity cell cycle identifies most division (blue and orange) in the prePB and PB populations. (*D*) Multivariate model to compare differential gene expression profiles between combined LGI1- and CASPR2-reactive cells versus -unreactive cells from both B cells and ASCs. This identified 25 genes up-regulated in the LGI1- or CASPR2-reactive population, and none which were down-regulated. (*E*) Differential gene expression data for each patient show cell type–specific upregulation of these 25 genes, and gene set enrichment analysis revealed that they associate with the term “positive regulation of intrinsic apoptotic signaling pathway”.

Next, we leveraged the scRNA-seq data to study the intrathecal differentiation of these CSF cells. First, we compared CSF B-lineage gene expression profiles to in vitro generated memory B, preplasmablast, plasmablast, and plasma cells (29). Overall, the LGI1- and CASPR2-reactive B cells and ASCs showed more differentiated gene expression patterns (Fig. 3*A*): 3/4 CD20^+^ LGI1- or CASPR2-reactive B cells were classified as preplasmablasts and expressed the canonical plasma cell gene XBP1, by comparison to 1/10 LGI1- and CASPR2-unreactive B cells (Fisher’s exact test, *P* = 0.04, Fig. 3 *A* and *B*). Other established markers of plasma cells (IRF4, BLIMP-1 and CD27, or loss of HLA-DR) did not separate autoantigen-reactive and unreactive B cells (*SI Appendix*, Fig. S3*A*). Within ASCs, 5/131 (4%) were classified as preplasmablasts, 89/131 (68%) as plasmablasts and the remaining 37 (28%) as plasma cells (Fig. 3*A*). Of these, 73/89 (82%) plasmablasts compared to 36/37 (97%) plasma cells were LGI1- or CASPR2-reactive (Fisher’s exact test *P* = 0.02, Fig. 3*A*). Overall, by comparison to LGI1- and CASPR2-unreactive ASCs, the LGI1- and CASPR2-reactive ASCs were more differentiated. The directionality of this differentiation was studied with RNA velocity analysis which revealed a dominant velocity stream from the actively dividing plasmablasts toward nonproliferative plasma cells (Fig. 3*C* and *SI Appendix*, Fig. S3 *B* and *C*).

In these, and other autoantigen-specific, syndromes, one ideal precision therapy might exclusively target the autoantigen-reactive CSF B-lineage cells. To this end, we further exploited the RNA sequencing datasets to perform a multivariate transcriptional analysis which incorporated LGI1 or CASPR2 reactivity and the differentiation state, treating each individuals’ cells as a pseudobulk sample. From this, 25 genes were identified, whose expression was significantly up-regulated and expressed >100-fold more in LGI1-and CASPR2-reactive B cells and ASCs, compared to their nonreactive counterparts (Fig. 3*E*). Gene ontology networks associated with these genes, displayed the term “positive regulation of intrinsic apoptotic signaling pathway”. No genes were down-regulated. Hence, by comparison to their unreactive counterparts, both the LGI1- and CASPR2-reactive B cells and ASCs exhibited features consistent with more active differentiation, particularly from plasmablasts to plasma cells, and showed up-regulated genes which mainly related to apoptotic pathways.

### Intrathecal Versus Peripheral Contributions to CSF BCR Diversity and Affinity.

**Fig. 4.**
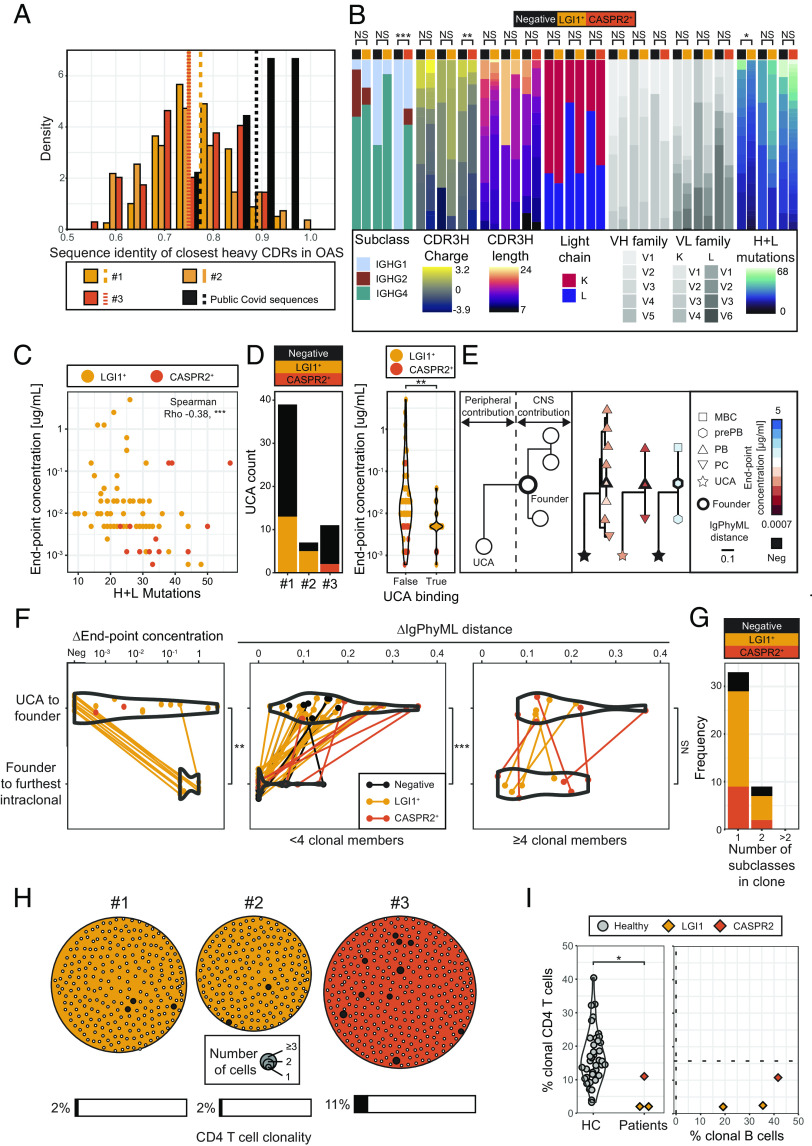
BCR characteristics and relative intrathecal versus peripheral contributions to mutational distances and autoantigen binding strengths. (*A*) LGI1- and CASPR2-reactive antibodies from all three patients are highly private when referenced against the OAS database. Contextualized against the closest heavy CDR sequence identities to OAS for nine clonal representatives from public SARS-CoV-2 ASCs (black). Mean values shown with vertical lines. (B) Within individual patients, by comparison to LGI1- and CASPR2 nonreactive BCRs, the LGI1- and CASPR2-reactive BCRs showed few consistent statistically significant differences. Pooled data shown in *SI Appendix*, Fig. S4*A*. (*C*) Monoclonal antibodies (mAbs) with lower end point concentrations (higher binding strengths) have more heavy plus light chain mutations (linear regression, Spearman’s R^2^ = −0.38, *P* < 0.0001; R^2^ = −0.35 for LGI1 and R^2^ = −0.27 for CASPR2). One mAb per clonal group was included. (*D*, *Left*) Overall, 20/57 (35%) of UCAs bound either LGI1 or CASPR2 on live cell-based assays and (*Right*) those which bound LGI1 or CASPR2 as UCAs overall showed higher end point concentrations as mutated CSF mAbs (*P* = 0.002, Mann–Whitney test). (*E*) Illustration of how characteristics of UCA-versus-founder and founder versus intraclonal members were used to infer relative peripheral and CNS contributions. End point concentrations are consistent within clonal groups which show some differentiation. Colors represent end point concentration of mAb, shapes represents cell type [memory B cells (MBC), preplasmablasts (prePB), plasmablasts (PBs), plasma cells (PCs), and unmutated common ancestors (UCA)], line length represents IgPhyML distance, and black outline highlights the designated founder BCR within the clonal group. Three clonal groups are shown. Others are in *SI Appendix*, Fig. S4*C*. (*F*) Comparisons of differences between log end point concentrations (*Left*) and mutational distances (*Middle* and *Right*; IgPhyML-generated arbitrary distances between BCR sequences) (32) for the UCA to founder intrathecal BCR versus the founder to the furthest clonal member for each tested clonal group. 17 of 28 (61%) UCAs begin as negative for LGI1 or CASPR2 reactivities (*Left*) and overall show a significantly higher change in end point concentrations versus the intraclonal range (*P* = 0.004). In smaller clones (<4 members, there was a significant difference between the UCA to founder phylogenetic Ig sequence distances (n = 34) versus intraclonal members (n = 34; *P* = 0.0001, Wilcoxon signed-rank test), but not within the nine clones with ≥4 members (*P* = 0.24, Wilcoxon signed-rank test). (*G*) 33/42 (79%) of clones have BCRs which only show one subclass, 9/42 express two subclasses and none >2 subclasses per clone. (*H*) Data from the three studied patients showing all isolated clusters of clonally related CD4^+^ T cells. Each dot represents a CD4^+^ T cell, and its size represents the number of clonal members. 2 to 11% of total CD4^+^ T cells (6/242 (2%) patient #1, 4/193 (2%) patient #2 (both gold), and 37/343 (11%) patient #3, orange) lie within clones of >1 member. (*I*) Percentage of clonally related CD4^+^ T cells and B cells for the two patients with LGI1 antibodies and one with CASPR2-antibodies. Dotted lines demonstrate clonally related CD4^+^ T cell (n = 54,267 cells from 41 donors) and B cell (n = 8 cells from three donors) frequencies from referenced healthy control datasets (21, 33).

These findings indicated that LGI1- and CASPR2-reactive cells were dividing and differentiating in the CNS, potentially upon exposure to their cognate autoantigens. To gain insights into the processes underlying maturation of these ASC-dominated intrathecal clonal expansions, BCR characteristics were explored. First, we investigated how individualized (“private”) the LGI1- and CASPR2-reactive BCR sequences were, by evaluating the closest heavy complementarity-determining region (HCDR) identity of each BCR to over 2 billion natural human heavy chain sequences in the Observed Antibody Space (OAS) database (30). Most LGI1- and CASPR2-reactive BCRs appeared highly private, with 61 to 67% lying at a closest HCDR sequence identity to OAS of <80% (Fig. 4*A*). In contrast, only 1/9 (11%) from a dataset of known public ASC-derived BCRs (31) fell in this region (Fig. 4*A*). Next, core characteristics of these private BCRs were compared to LGI1- and CASPR2-unreactive BCRs (Fig. 4*B* and *SI Appendix*, Fig. S4*A*). This revealed few differences at the individual patient level across Ig subclass, CDR3 length/charge, light chain usage, VH/VL family gene usage and total BCR mutations (Fig. 4*B*) Yet, in a pooled analysis, the autoantigen-reactive BCRs were more commonly of the IgG4 subclass (*P* = 0.003, Fisher’s exact test), with CDR3H sequences that were more negatively charged (median of −1 versus 0; *P* = 0.006, Mann–Whitney) and shorter (median of 13 versus 15 amino acids; *P* = 0.004, Mann–Whitney) (*SI Appendix*, Fig. S4*A*). Of all these, total heavy- plus light-chain mutations was the BCR characteristic which most closely correlated to lower autoantibody end point concentrations, a surrogate of greater BCR and secreted antibody binding strength (Spearman’s R^2^ = −0.38; *P* < 0.0001; Fig. 4*C*).

Next, we asked whether these BCR mutations, and the commonly associated property of affinity maturation, were predominantly acquired in the periphery or intrathecally. Understanding these relative contributions fundamentally influences which compartment to consider as an optimal therapeutic target, and, additionally, evidence of intrathecally generated diversity implicates CNS-based TLS in the pathogenesis of these diseases. To assess purely CNS contributions, BCR mutations and affinities were compared between the intrathecal founder of each clone (defined as the least mutated observed clonal member) and its most distant intraclonal member. To estimate peripheral contributions, the same intrathecal founder was compared to its unmutated common ancestor (UCA), the BCR which represents its naive, germline precursor (Fig. 4 *D*–*F*).

Overall, 65% of UCAs did not bind LGI1 or CASPR2. Yet, 35% retained binding to their autoantigen, typically those with stronger binding original “parent” mAbs (Fig. 4*D*, *P* = 0.002, Mann–Whitney test; *SI Appendix*, Fig. S4 *B* and *C*). Hence, germline LGI1 or CASPR2 reactivity is a feature of some CSF BCRs. Next, we studied intrathecal affinity maturation. Despite observed examples of cellular differentiation from any of memory B through preplasmablast, plasmablast, and plasma cells (Figs. 3 *A*–*C* and 4*E* and *SI Appendix*, Fig. S4*D*), it was striking that no tested lineage trees showed intraclonal members with improved end point concentrations. Overall, the diversity of intraclonal end point concentrations was markedly restricted, and around three log-folds lower than the marked differences frequently present between UCA and the founder intrathecal BCR end point concentrations (*P* = 0.004, Mann–Whitney test; Fig. 4*F*). Consistent with this, a comparison of heavy chain immunoglobulin sequence distances (ΔIgPhyML) (32) between these two cellular populations showed limited overall sequence differences within members of CSF clonal expansions (range 0 to 0.15; median 0) by comparison to far greater sequence distances between UCAs and the founder clonal member (0.03 to 0.36; median 0.15; *P* < 0.0001, Wilcoxon signed-rank test; Fig. 4*F* and *SI Appendix*, Fig. S4*D*). However, the few larger, and exclusively autoantigen-reactive, clones with ≥4 CSF members showed greater intraclonal sequence diversification, comparable to that of their UCA-to-intrathecal founder distances (Fig. 4*F*). Further, another typical function of GCs, IgG subclass switching, was only observed in 9/42 (21%) CSF clones, and no clones expressed >2 subclasses (Fig. 4*G* and *SI Appendix*, Fig. S4C). Collectively, these findings suggest that most LGI1- and CASPR2-reactive clonal ASC expansions exhibit limited intrathecal BCR hypermutation or class switching, and none associate with improved affinity toward their autoantigen.

Given this, we asked whether this limited intrathecal maturation was accompanied by minimal contributions from CD4^+^ helper T cells. Consistent with this hypothesis, CSF TCR analyses from these three patients showed low proportions of clonally expanded CD4^+^ T cells (2, 2 and 11% from a total of 778 CD4^+^ cells, Fig. 4*H*), by comparison to healthy control CSFs (Fig. 4*I*; *P* = 0.02 Wilcoxon test) (33). Further, these expansions were not proportional to the corresponding frequencies of B-lineage clonal expansions within individual patients (Fig. 4*I*). Taken together, small clonal B-lineage expansions were accompanied by limited class switching and few T cell clones which contrasts strikingly to the profound increases in affinity and mutational distances between UCA and founder clones.

## Discussion

By comprehensively reconstructing the human CSF BCR repertoire from patients with prototypical autoantigen-mediated CNS diseases, our data identify clonally expanded ASCs as a remarkably frequently autoantigen-reactive population. To our knowledge, this represents the most enriched autoantigen-reactive lymphocyte compartment throughout medicine, most notably the 100% rates observed within larger clonal expansions. This autoantigen-reactive ASC population showed intrathecal differentiation from plasmablasts to plasma cells, and, by comparison to nonreactive counterparts, upregulation of genes associated with apoptosis signaling pathways. Yet, despite evidence of differentiation within the CSF, these cells had acquired few intrathecal mutations without affinity maturation toward LGI1 or CASPR2 and were not accompanied by an increased frequency of clonal CD4^+^ T cells. In contrast, marked differences between UCAs and their CSF founders indicate that peripherally driven, likely GC dependent, processes make significantly greater contributions to the overall affinity maturation of LGI1- and CASPR2-reactive B-lineage cells. Taken together, while autoantigen-reactive B cells show some distinctive markers which could represent specific therapeutic targets, our data suggest a limited role for the CNS in maturation of likely pathogenic ASCs. Rather, targeting of the more biologically influential peripheral compartment in these conditions may represent a more robust therapeutic strategy.

A dominant peripheral contribution to BCR maturation is also consistent with the several-fold higher autoantibody levels in serum versus CSF of AE patients (1, 10, 11) and is supported by the detection of circulating LGI1-reactive memory B cells with BCR mutations and end point concentrations comparable to those of our CSF-derived BCRs (7). The peripheral GCs most likely to mediate this maturation are situated within deep cervical lymph nodes, which drain CNS lymphatics and represent a site of preferential autoantibody synthesis in other neurological autoantibody-mediated diseases (34, 35). Our observations from UCAs suggest that these peripheral GC reactions are typically initiated by naive B cells with undetectable reactivities against LGI1 or CASPR2. However, as the subsequent somatic hypermutations, affinity maturation, and class switching are very likely GC- and T cell–dependent processes (25), it remains difficult to resolve the reported absence of LGI1-reactive T cells in the blood of these patients (36). This represents an area for future consideration.

Intrathecally, the limited BCR mutational activity was accompanied by no examples of improved affinity and minimal intraclonal class-switch recombination. Therefore, collectively, our findings indicate an absence of intrathecal GC activity and hence CNS-resident TLS. This conclusion is more indirectly supported by the low observed frequency of clonal CSF CD4^+^ T cells, and the histological absence of TLS despite very few LGI1 or CASPR2 antibody encephalitis brain specimens having been studied (37). Whereas the meningeal TLS observed in multiple sclerosis are proposed to mediate intrathecal somatic hypermutation and CSF-compartmentalized progressive disease (26), the absence of such structures in patients with LGI1 and CASPR2 antibody encephalitis, including one with a long untreated history, may explain their more monophasic course without progression.

Therefore, we hypothesize that the observed intrathecal clonal ASC bursts may be largely independent of cognate T cell help and GCs, perhaps more likely to be mediated by BCR sensing of soluble cognate autoantigens in the CSF (7, 38). The upregulation of proapoptotic genes suggests exposure to these autoantigens may be driving the ASCs toward death, perhaps utilizing a post-GC-based mechanism to eliminate autoreactivity (39). Nevertheless, these bursts overall generate frequencies of autoantigen-reactive B-lineage cells which surpass those of the highest reported to date, in the gut of patients with celiac disease (40).

Our data also suggest that soluble serum autoantibodies crossing blood–brain barriers make a limited contribution to disease pathogenesis. The 100% autoantigen specificity rate in larger CSF clones and, still remarkable, 62% frequency within singletons strongly supports the necessity for intrathecal ASCs actively secreting LGI1 and CASPR2 antibodies in immediate proximity to their target organ. Yet, peripherally generated soluble autoantibodies entering the CNS cannot be formally excluded by our observations.

In addition to highlighting the underlying biology, our findings have potential implications for future therapeutic trial designs. For example, given many intrathecal ASCs are plasmablasts, which are not considered targets of bortezomib (41), this drug may be ineffective in AE. By contrast, the universal expression of CD19 on intrathecal LGI1- and CASPR2-reactive ASCs suggests targeting this molecule may hold greater promise. Also, they help confirm the presence of intrathecal lymphocytes despite normal routine CSF cell counts (10).

Limitations of our study include that only three patients were sampled. However, unlike previous CSF studies in AE (10, 17, 18), they received no or very minimal immunotherapies, more faithfully reflecting the fundamental immunobiology. Further, our intention was to compensate for low patient numbers with analysis depth: 166 patient-derived autoantigen-reactive mAbs represent a substantial dataset for any molecular autoantibody study across medical disciplines, and the Smart-seq2 methodology generated four-fold more unique transcripts than previous droplet-based methods using CSF (21, 42). Nevertheless, larger studies are required to establish whether differences exist between LGI1 and CASPR2 antibody patient CSFs, and in other neuroinflammatory diseases. Our evaluation of UCAs to infer peripheral contributions is justified because it focused our study on the key BCRs isolated from the CNS and is a widely acknowledged approach to infer germline reactivities (43, 44). However, we did not directly isolate unmutated autoantigen-reactive cells from the peripheral blood or bone marrow, as this is likely to require a different workflow which may be technically challenging as both their frequencies and affinities are likely very low (10, 45). Also, despite sampling >10% of the human CSF compartment, we observed few autoantigen-specific CSF memory B cells, meaning trajectories could not be inferred across all B-lineages. To explain this, we hypothesize memory B cells rapidly differentiate within the autoantigen-rich CNS into the more abundant ASCs. Finally, the extent of intrathecal immunoglobulin class-switch recombination should be compared to non-IgG4 autoantibody-associated diseases as further IgG recombination cannot be studied after expression of the most downstream constant gene encoding IgG4.

In summary, our data suggest that CNS-based processes play limited roles in the differentiation, somatic hypermutation, and affinity maturation of the autoantigen-reactive CSF ASCs which are ultraenriched in patients with LGI1 and CASPR2 antibody encephalitis. These findings should direct key biological studies and therapeutic interventions to the peripheral immune processes in these diseases.

## Materials and Methods

### Participants and CSF Antibody Determination.

The study was approved by the Yorkshire and Humber Research Ethics Committee (REC16/YH/0013), and participants gave written consent. Throughout, data for the two patients with LGI1 antibody encephalitis are shown in gold, whereas data for the CASPR2-antibody encephalitis patient are shown in orange. CSF (12 mls) was obtained from three patients (Fig. 1*A*), pelleted for 10 min at 500 G, and stained as below. To measure levels of total LGI1-IgG and IgG1-4 subclasses, the soluble CSF fraction was incubated with live HEK293T cells stably transfected to express membrane-tethered LGI1-EGFP and binding by subclass-specific secondary antibodies quantified with flow cytometry, as previously described (13). Total IgG in CSF and serum was quantified by laser immunonephelometry and an antibody index calculated as LGI1 or CASPR2-IgG [CSF/serum]/Total IgG [CSF/serum].

### CSF FACS.

The cell pellet was stained in ice-cold phosphate buffered saline (PBS) with 2% fetal calf serum (FCS) containing antibodies to CD3, CD4, CD8, CD14, CD19, CD20, CD27, CD38, CD138, plus 7AAD as a live-dead cell marker. Single-cell sorting utilized a Sony MA900 within 1 h from CSF collection. Indexed fcs files were compensated with FlowJo (Ashland, OR: Becton, Dickinson and Company; 2023), and all further analysis, including integration with transcriptomic data, was performed in R.

### scRNA-Seq with Smart-seq2.

Single-cell full-length transcriptome libraries were generated with the Smart-seq2 protocol-2019 update (27) with minor modifications (*SI Appendix*). These data have been uploaded to the European Genome-phenome Archive—EGAD00001006232. Other raw data within this study are available from the corresponding author on reasonable request.

### Bioinformatic Analyses.

Paired fastq files were generated with Illumina bcl2fastq software. Quasi-mapping and transcript abundance estimation was performed with Salmon (46), using Ensembl human coding- and noncoding RNA transcriptomes. BCR and TCR sequence determination and abundance estimation was performed with BraCer and TraCeR, respectively (47, 48). Clones were defined as sharing V-gene and J-gene segments with identical CDRH3 lengths and Hamming distance <6. The founder member of a clone was defined as that with the fewest mutations. Downstream quality control, filtering, and analyses were primarily performed within the OSCA framework (49), and BCR and TCR sequence data were analyzed with the Immcantation package suite (28). Further bioinformatic processing methods are detailed within *SI Appendix*. The code workflow and the data to reproduce the figures can be found at https://github.com/jtheorell/LGI1_CASPR2_BCR_CSF.

### BCR Cloning, UCA Generation, and mAb Binding Detection.

Amplified or synthesized BCR variable region sequences were cloned into heavy or light chain expression vectors corresponding to their native IgG subclasses, as previously described (50). To obtain UCA sequences, somatically hypermutated V(D)J sequences were aligned with IgBlast to identify the best-matched germline genes. Thereafter, sequences were backmutated by replacing nonsilent mutations with their corresponding germline template nucleotides. To avoid inaccurate alignments, N-region nucleotides were not modified as they are nontemplated random insertions at the gene segment junctions. All reverted sequences were then cloned into the corresponding heavy or light chain IgG expression vector, as above. mAb antibody binding was detected using live cell-based assays employing transiently-transfected HEK293T cells surface-expressing full-length LGI1, CASPR2 and AQP4, live cultured hippocampal neurons and fixed adult rat brain sections, as have been previously described (7, 8) and in *SI Appendix*.

## Supplementary Material

Appendix 01 (PDF)

## Data Availability

Code workflow and the data to reproduce the figures data have been deposited in Github (https://github.com/jtheorell/LGI1_CASPR2_BCR_CSF) (51). Some study data are available. We will upload our data to the European Genome archive (EGA). Access to uploaded data requires a request from interested researchers to be ethically approved by an internal committee. All our code has been uploaded to a Github page, as cited in the manuscript. All data are available in the main text, supplementary materials, or will be made available to researchers once data/material transfer agreements are in place.
