# Supplementary material for: Ultrahigh frequencies of peripherally matured LGI1- and CASPR2-reactive B cells characterize the cerebrospinal fluid in autoimmune encephalitis
Source: Proc Natl Acad Sci U S A. 2024 Feb 6;121(7):e2311049121. doi: 10.1073/pnas.2311049121 (PMC10873633; doi:10.1073/pnas.2311049121)
Supplement: Supplementary file 1 — Appendix 01 (PDF) [file pnas.2311049121.sapp.pdf]

Supporting information

Supplementary Table 1. Detailed clinical features of the three patients with LGI1 and CASPR2-antibody encephalitis.

	**Patient #1**	**Patient #2**	**Patient #3**
Age, sex	84, F	53, M	71, M
Days of symptoms prior to sample collection	21	42	1041
Seizure semiologies	FBDS*	FBDS*, sensory	Rising epigastric sensations
Cognitive features	Amnesia, Personality change	Amnesia, Personality change	Amnesia, Personality change
Other features	Nil	Nil	Ataxia
MRI features of encephalitis	Nil	Nil	Nil
CSF autoantibodies / oligoclonal bands (OCBs)	LGI1-antibodies positive / OCBs Negative	LGI1-antibodies positive / OCBs Negative	CASPR2-antibodies positive / OCBs Negative
CSF protein level (mg/mL; NR<0.6)	0.25	0.29	0.24
Leucocyte count (NR <5 / ml)	2	0	0
HLA-DRB1 allele	07:01	07:01	11:01
Immunotherapy	Corticosteroids (IV and PO), plasma exchange	Corticosteroids (IV and PO), plasma exchange	Corticosteroids (IV and PO), plasma exchange
Duration of immunotherapy prior to sample collection	Nil	One dose of IV corticosteroids	Nil
Total duration of immunotherapies	3 years	2 years	3 years
Addenbrooke's cognitive examination (/100) at onset and at 2 years (/day)	69 to 93	84 to 90	56 to 76
Seizure frequency at onset and at 2 years (/day)	28 to 0	75 to 0	20 to 0
Modified Rankin Score at onset and 2 years	3 to 1	3 to 1	4 to 2

*FBDS = faciobrachial dystonic seizures

Supplementary Figures

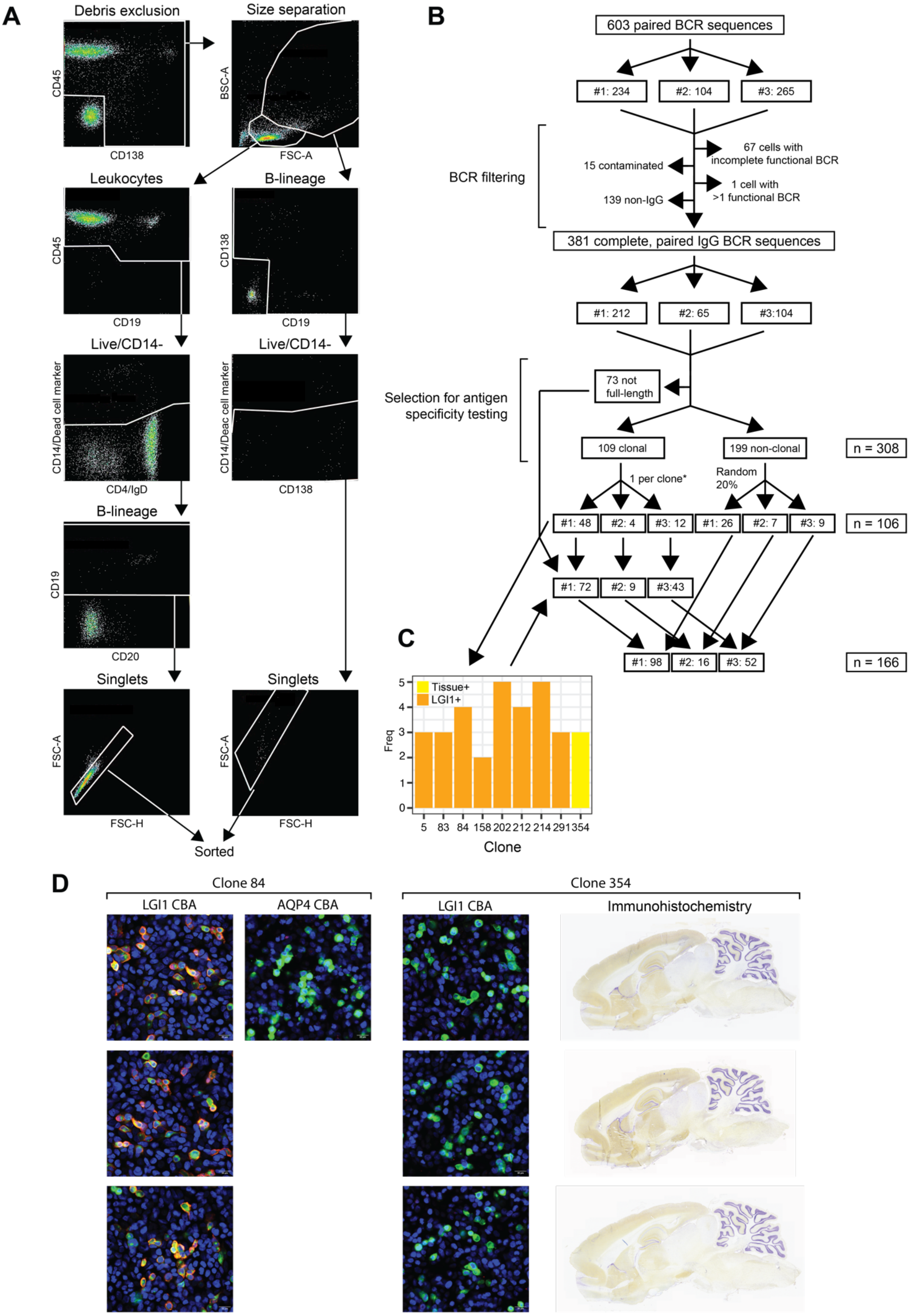

Supplementary Figure 1. Gating strategy, BCR quality control and cloning selection strategy. A. Sort strategy aimed to maximise capture of antibody-secreting cells, including those with low CD45 expression. First gate shows only $CD45^{-}CD138^{-}$ cells were excluded and, thereafter, due to different autofluorescence and scatter characteristics, two separate gating strategies were devised for cells with $scatter^{low}$ (lymphocyte; left column) and $scatter^{high}$ (other leukocyte; right column) characteristics incorporating exclusion of $CD4^{+}IgD^{+}CD14^{+}$dead cells (left column) or $CD14^{+}$dead cells (right column). After sorting 384 cells, further sorting was restricted to $scatter^{high}$ cells and $scatter^{low}$ $CD19^{+}$ cells. After doublet exclusion, cells from both columns were sorted together using a Boolean OR gate. Rare events have been obscured from images to remove annotations. **B.** Acquired BCRs went through quality control to ascertain complete, paired BCR sequences. Cells were excluded if they contained: i) identical but fewer light chain reads in neighbouring wells (contamination, n=15); ii) BCRs which were either incomplete (n=67) or showed >1 functional BCR (n=1); iii) non-IgG isotypes (n=139). From these, at least 1 per clonal group and a random 20% of non-clonal cells were selected for cloning and monoclonal antibody (mAb) expression. Cells were selected using a random number generator. Data are shown per patient (#1, #2 and #3) and combined. **C.** Expressed monoclonal antibodies representing members of nine tested clonal groups showed consistent intraclonal binding to either LGI1 (orange) or on immunohistochemistry (yellow, 'Tissue^{+}'). **D.** Representative positive (clonal group 84) and negative (clonal group 354) LGI1-binding shown. All three tested members of clonal group 354 were blindly identified to bind rodent brain sections with strikingly similar patterns. In cell-based assays, LGI1-tagged with EGFP colocalises with red AF594 goat anti-human Fc IgG specific staining on live cell-based assays (DAPI in blue). Results were only deemed LGI1- or CASPR2-reactive if mAbs did not bind to live cell-based assays expressing aquaporin-4

(AQP4; AQP4-tagged with EGFP in green). These results show why a single member of each clone could be tested and reliably assumed to show an antigenic-specificity representative of its clonal group.

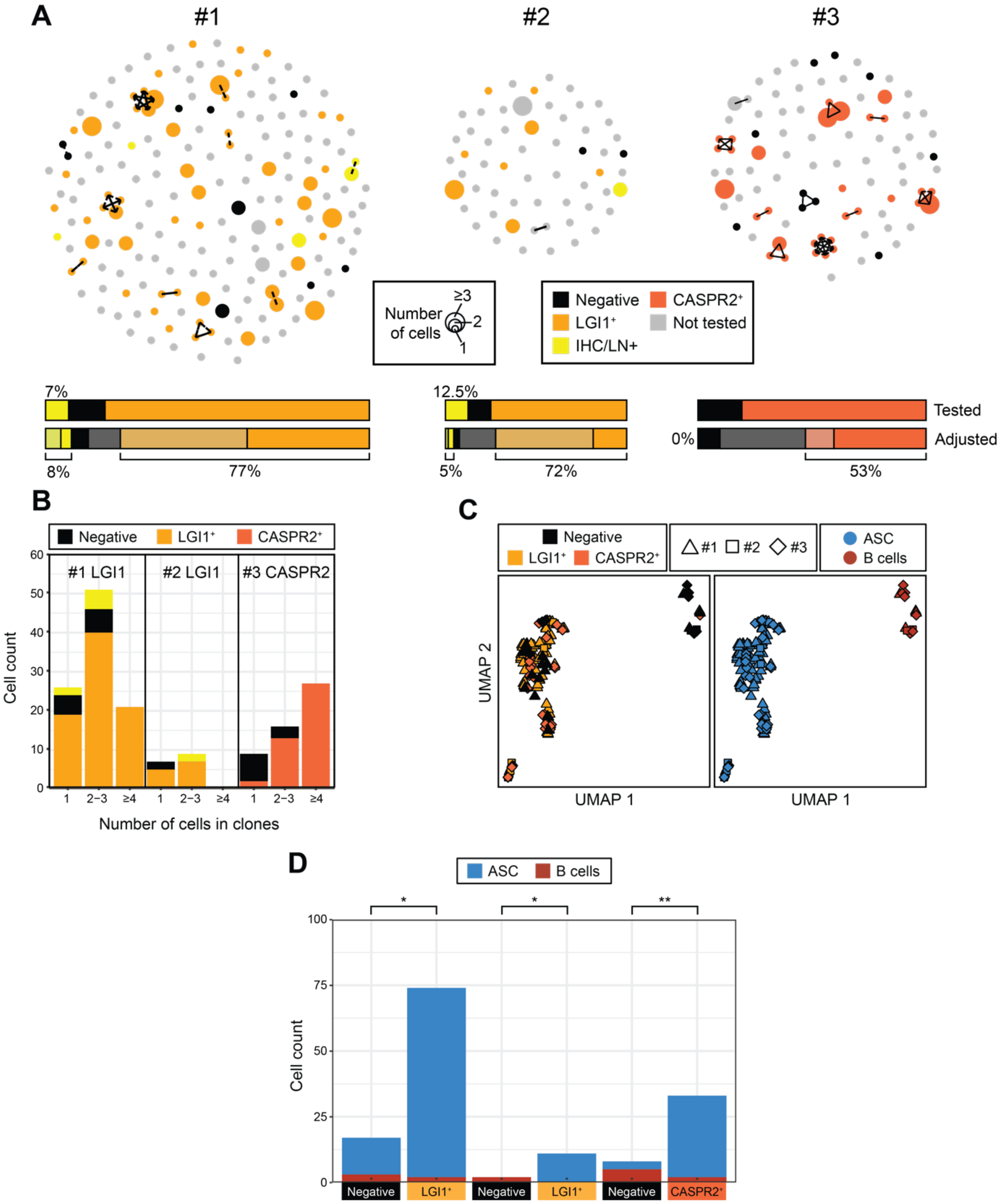

Supplementary Figure 2. LGI1 and CASPR2 and broader antigenic reactivities of patient-derived mAbs. A. Data from Fig.2A showing all clusters of clonally related immunoglobulin heavy chain variable regions in the three patients. Each dot represents one heavy chain, and

its size represents the number of times that heavy chain sequence was observed; two dots connected by a line are clonally related; dot colour represents specificity where gold is LGI1-, orange is CASPR2-reactive, yellow represents immunohistochemistry (IHC) and/or live neuron binding and black is no reactivity to LGI1, CASPR2, IHC or neuron binding. Grey dots show those which were not expressed as mAbs. Specificity is assumed to be consistent within clonal groups. **B.** LGI1 and CASPR2-specificity (from Fig.2B) extended to include IHC and/or live neuron binding based on clonal sizes (1, 2-3 and ≥4). **C.** UMAP from Fig.2C to incorporate data from individual patients. **D.** Data from Fig.2C divided by individual donors (Fisher's exact test p=0.043, p=0.012 and p=0.001 for the three patients).

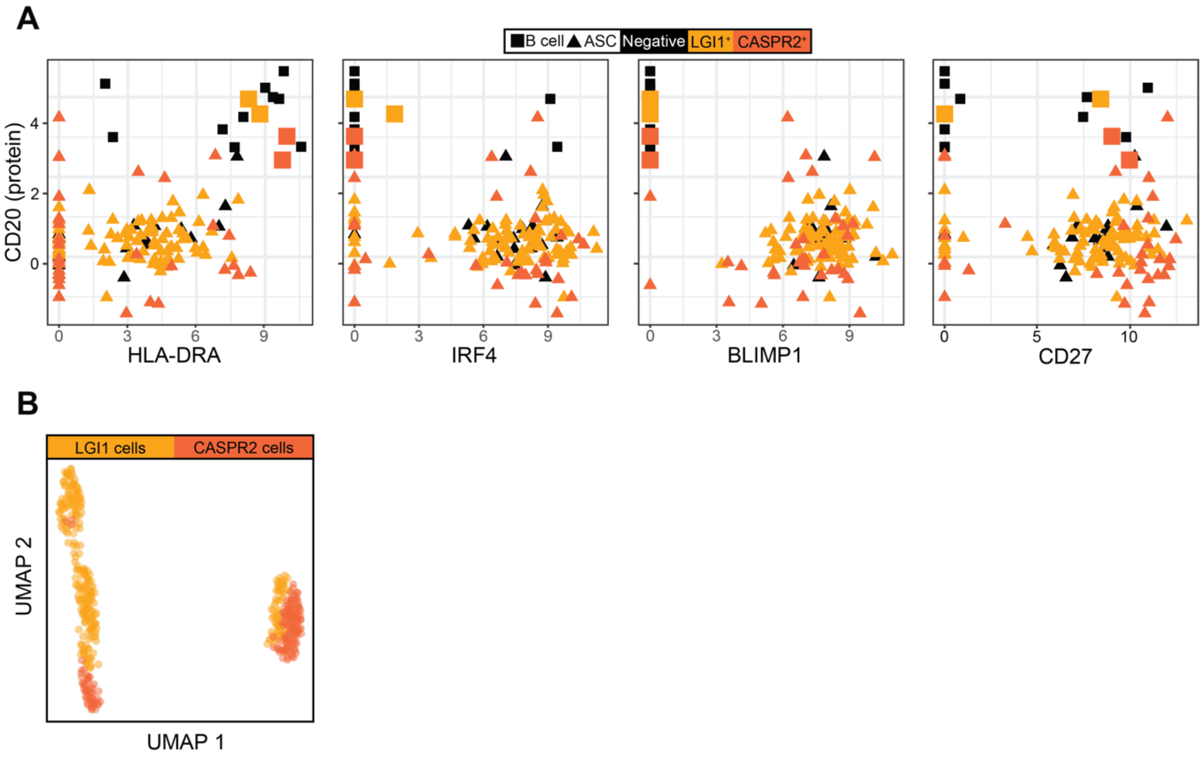

Supplementary Figure 3. Expression of canonical ASC markers in LGI1 and CASPR2-reactive B cells. A. 4/4 LGI1 or CASPR2-reactive B cells express CD20 protein and HLADRA mRNA, 1/4 express increased IRF4 RNA, none express BLIMP1 RNA and 3/4 express high levels of CD27 RNA. Colour represents specificity, shape represents B-lineage subset and LGI1/CASPR2-reactive B cells are represented in a larger size for visualisation. **B.** RNA velocity data divided for cells from LGI1- (gold) and CASPR2- (orange) antibody patients.

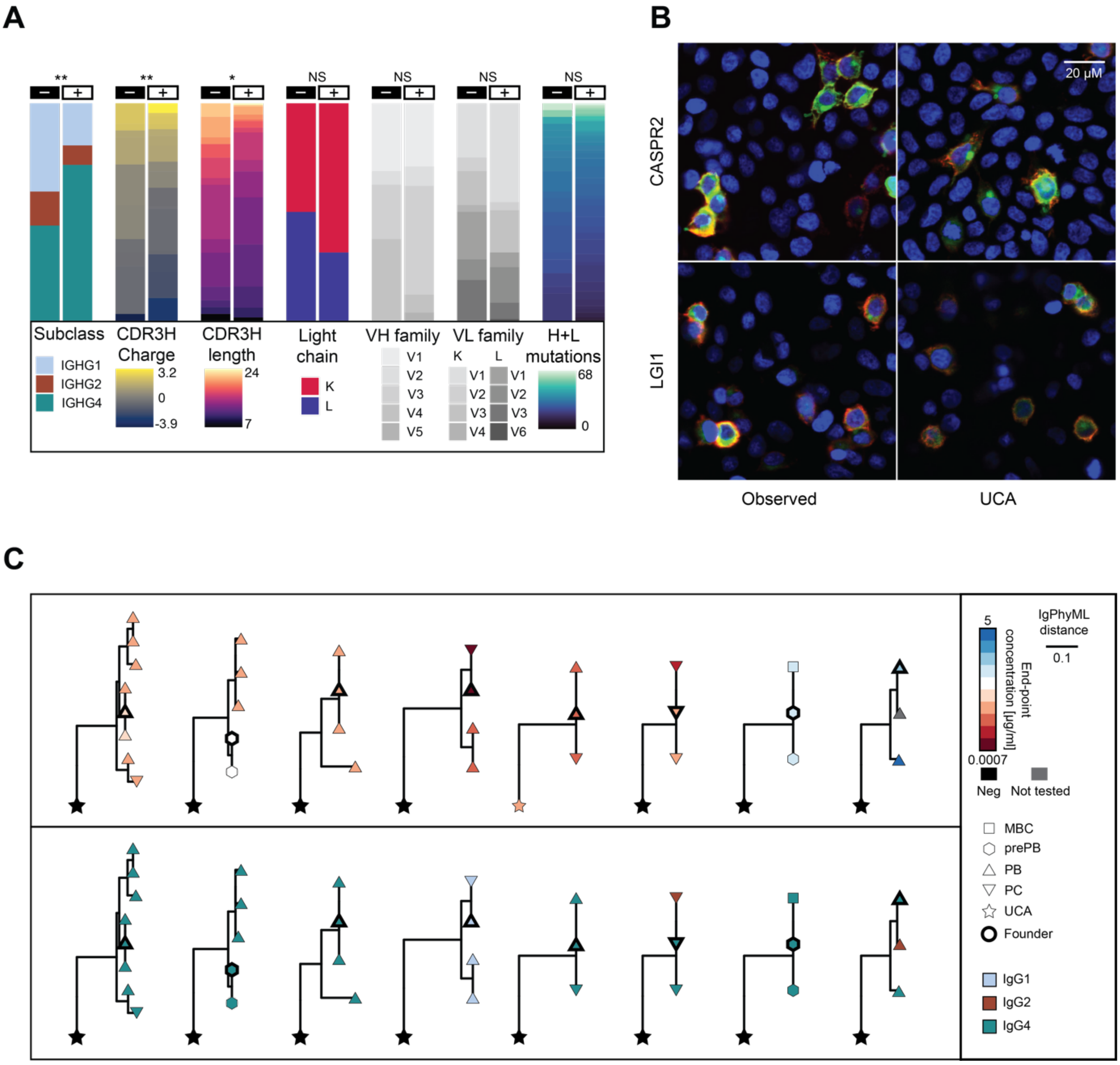

Supplementary Figure 4. BCR characteristics and intrathecal clonal trees. A. Pooled analysis of LGI1 and CASPR2-reactive (n=132) versus unreactive (n=34) BCR comparisons across parameters from Fig.4B shows more IgG4s (Fisher's exact test, IgG4 vs non-IgG4, p=0.004) with have more positively charged (Mann-Whitney U test, p=0.017) and longer heavy chain CDR3 regions (Mann-Whitney U test, p = 0.006) in the autoantigen-reactive population, without significant differences in light chain usage (Fisher's exact test, p = 0.06), VH family and VL usage (ANOVA, p = 0.12 and 0.098, respectively) or heavy plus light chain mutation frequencies (Mann-Whitney U test, p = 0.06). **B.** Cloned and expressed monoclonal

antibodies (mAbs) from unmutated common ancestors (UCAs) were tested on live cell-based assays to determine LGI1 or CASPR2 specificity (representative binding of UCAs and their paired mutated mAbs are shown; DAPI staining in blue, LGI1 or CASPR2-tagged with EGFP colocalises with red AF594 goat anti-human Fc-IgG). **C.** mAb endpoint concentrations show consistency within clonal groups but intraclonal variation. Dot colour represents endpoint dilution (blue through red; black = negative), dot shape represents the cell type (as Fig.4E: memory B cells (MBC), preplasmablasts (prePB), plasmablasts (PBs), plasma cells (PCs) and unmutated common ancestors (UCA)), line length represents IgPhyML distance and black outline highlights the observed BCR which is the designated founder of the clonal group. All clonal groups which were tested for endpoint concentration are shown. IgG subclasses shown in light blue (IgG1), red (IgG2) and green (IgG4).

Methods

Smart-seq2

Smart-seq2 (1) modifications included removal of the bead cleanup step after the RT-PCR steps, as well as tagmentation of the raw PCR product, changes that were introduced after personal communication with Dr Simone Picelli, first author of the original protocol. All reactions were performed in 384 microwell PCR plates. Dispensing was performed with a Formulatrix Mantis and nano-pipetting was performed with an SPT Labtech Mosquito LV. Sequencing of paired 74 base length fragments was performed on an Illumina NextSeq instrument with NextSeq v2.5 150 cycle high output kits. ERCC spike-ins (Thermo-Fisher) were used for normalisation.

mAb binding to LGI1, CASPR2 and other neuronal substrates

For cell-based assays, mAbs were incubated from 20 µg/ml with the extracellular domains of LGI1, CASPR2 and AQP4 transiently surface expressed on live HEK293T cells, all C-terminally fused to enhanced green fluorescent protein (EGFP), as described previously.(2) Next, transfected HEK293T cells were fixed with 4% formaldehyde, and visualised with Alexafluor 594 conjugated anti-human IgG Fc antibodies (709-585-098; Jackson Labs). Endpoint dilutions were defined as the final dilution at which binding was visible. Live cultured hippocampal neurons at day *in vitro* 25 and 4% formaldehyde fixed adult rat brain sections were also incubated with LGI1/CASPR2-unreactive mAbs and visualised, as previously described.(3)

Bioinformatics workflow

Raw data handling, generation of BCR/TCR sequences, generation of RNA velocity data up to and including the velocyto analyses were done in Unix shell script. These analyses were conducted utilizing high-capacity cloud computing services, connected to the MRC WIMM Centre for Computational Biology, the National Academic Infrastructure for Supercomputing in Sweden (NAISS) and the Swedish National Infrastructure for Computing (SNIC) at Uppsala Multidisciplinary Center for Advanced Computational Science (UPPMAX). Below, all programming was conducted in R (4), unless otherwise specified.

Flow cytometry analyses

The indexed flow cytometry data were compensated using a FlowJo (Becton Dickinson, Franklin Lakes, New Jersey). A frozen standard PBMC control was used in each experiment for batch normalization, which was performed using flowSpecs (5) and flowCore (6). IgD and CD4 were acquired in the same channel on the Sony MA900 sorter, and were bioinformatically separated, based on the assumption that all $CD19^{+}$ cells are $CD4^{-}$ and all IgD^{+} cells are $CD19^{+}$. In protein-based visualization UMAPs for figure 2, CD138 was given a relative weight of 2, due to its significance in the separation of ASC from B cells.

Cell type definitions

Cell types were defined by index sort surface phenotype (SI Appendix Fig 1A). After exclusion of doublets, debris, dead cells and myeloid cells during the sort, $CD3^{+}CD138^{-}$ cells were defined as T-cells, $CD3^{-}CD138^{+}$ cells were defined as ASCs and $CD3^{+}CD138^{+}$ cells were defined as doublets. $CD3^{-}CD138^{-}CD19^{+}$ cells were defined as B-lineage cells: within this population, those which were $CD20^{-}CD38^{+}$ were defined as ASCs, whereas all others were defined as B cells. This gating strategy was verified using the SingleR package (7) and the Monaco dataset (8), with no B cells being SingleR-classified as ASCs and only 16 ASCs being classified as exhausted B-cells, all of which expressed CD138.

Raw data handling

BCL to fastq conversion was done with bcl2fastq (Illumina, San Diego, CA, USA). Trimming was performed with TrimGalore (9). For transcript definition and quantification, salmon was used (10) with Ensembl human coding- and non-coding RNA transcriptomes as well as ERCC standard transcripts included. Specific flags included in the analysis were “seqBias”, “validateMappings” and 4 rangeFactorizationBins. Conversion of Salmon results to a gene abundance matrix was performed with the R package tximport (11). For quality control of raw, trimmed and quasi-mapped files, fastqc (12) and multiqc (13) were utilized.

Generation of BCR and TCR sequences

To generate the BCR and TCR sequences, BraCeR (14) and TraCeR (15) were utilized on the trimmed fastq files, respectively. Standard settings for human samples were used for these analyses.

Generation of RNA velocity and cell cycle information

To generate the RNA velocity and cell cycle information, the trimmed fastq files were first analysed with STAR (16) to generate SAM files. Here, the intronMotif option was used for the outSAMstrandfield flag, as the data is unstranded. These were then analysed with velocyto (17) to create input data for scVelo (18). Velocyto was used with standard settings. Finally, the cell cycle algorithm in scVelo was used to define the cell cycle phase for each cell individually. scVelo was used in a python environment. For the scVelo filtering and normalization, 12000 genes was used, as the goal was to catch genes involved in the cell cycle and the sequencing run was very deep, allowing for this high number. A minimal shared count of 20 was also used as a cutoff. The "moments" function was used with 30 principal components and 30 neighbors. scVelo was run in a dynamical mode.

Transcriptomic analyses

Transcriptomic analyses, post tximport, were conducted primarily within the R OSCA framework (19). Specifically, packages ensembldb (20), SingleCellExperiment (19), Scran (21) and Scater (22) were used for metadata import, quality controls and visualisations. Cell filtering was performed with manual settings, due to the considerable difference in transcriptome size between B cells and ASCs, as well as a donor/batch difference in ERCC spike in fraction. 9% were filtered out due to low transcript count, few unique transcripts, and/or high percentages of mitochondrial transcripts and/or ERCC spike ins.

Transcriptomic comparison between AE B-lineage cells and in vitro-differentiated B-lineage cells

To further define the differentiation status of B-lineage cells, the *in vitro* plasma cell differentiation bulk RNA datasets from Kassambara *et al* (23) were used. Here, memory B-cells, pre-plasmablasts, plasmablasts and plasma cells were used as input to a SingleR (7) model which was then used to classify each single cell in the AE dataset into the one of these four categories which it most closely resembled.

BCR analyses

The Immcantation framework was primarily used for analysis, with the package Alakazam (24) providing core R functionality and together with Change-O used for clonality analyses

(24). For clonal definitions, the standard Hamming-based Changeo distance metric was used, in combination with a density-based threshold and a binwidth of 0.02. Change-O was called through a Unix shell script. Shazam (24) was used for mutation analyses and the full-length receptors, including the CDR3 and the FWR4 were used for these calculations. Dowser (25), in combination with the IgPhyML (26) package were used to create and visualize clonal trees. IgPhyML was used through a Unix shell script-called Docker image (27) to get correct R package dependency setup.

Public versus private BCR repertoire analysis

Each of the LGI1/CASPR2-reactive antibodies were probed against *c*. two billion human repertoire antibodies from the Observed Antibody Space database using KA-search (28, 29), returning the closest heavy CDR (IMGT definition (30)) sequence identity to the query. When profiling the reference set of nine ASC-derived public SARS-CoV-2 antibodies (31), OAS sequences from the repertoires of COVID-19 patients were omitted from the search to generate an internal reference repertoire, as there were no available sequences in OAS from AE patients.

Clonal tree distance calculations

To create a common statistic for the germline to the founder clone BCR distance and the intraclonal distances, the IgPhyML (26) algorithm were used. Here, the tree distance from the germline sequence to the least mutated observed CSF BCR sequence (founder), was calculated, as well as the longest intraclonal distance from this designated founder.

Public TCR data analyses

For the public data, 54,267 quality-controlled cells from 45 healthy individuals were studied (32). The corrected and filtered GEX matrix was merged and integrated using SCTransform with Seurat v4.1.2. Based on the same set of markers used by the authors, it was possible to resolve the cell clusters into their respective cell types. TCR clonality analysis was performed on the $CD4^+$ cluster for the 41 healthy controls with TCR data (excluding 4 healthy controls without matching TCR data). Frequencies for TCR clones > 1 cell for each of the healthy control was calculated with respect to the total $CD4^+$ cells for that individual.

Visualisations

The R Tidyverse package ggplot2 (33) was central to visualization. The ggplot2-associated packages ggforce (34) and ggnetwork (35) were also used, as well as DepecheR (36). Heatmaps have been generated with the package pheatmap (37).

Statistics and data presentation

For calculations of ANOVA, Wilcoxon signed ranks test and Wilcoxon rank sum tests (also known as Mann-Whitney U test), Fisher´s exact test, as well as correlation analyses, the R stats package (4) was used. For calculation of odds ratios, the epitools package (38) was used. Adobe Illustrator and BioRender were used for statistical analysis and data presentation.

References

1. S. Picelli, *et al.*, Full-length RNA-seq from single cells using Smart-seq2. *Nat. Protoc.* **9**, 171–181 (2014).

2. M. Ramberger, *et al.*, Distinctive binding properties of human monoclonal LGI1 autoantibodies determine pathogenic mechanisms. *Brain J. Neurol.* **143**, 1731–1745 (2020).

3. S. R. Irani, *et al.*, Antibodies to Kv1 potassium channel-complex proteins leucine-rich, glioma inactivated 1 protein and contactin-associated protein-2 in limbic encephalitis, Morvan's syndrome and acquired neuromyotonia. *Brain J. Neurol.* **133**, 2734–2748 (2010).

4. R Core Team, R: A Language and Environment for Statistical Computing (2017).

5. J. Theorell, flowSpecs: Tools for processing of high-dimensional cytometry data. (2022).

6. F. Hahne, *et al.*, flowCore: a Bioconductor package for high throughput flow cytometry. *BMC Bioinformatics* **10**, 106 (2009).

7. D. Aran, *et al.*, Reference-based analysis of lung single-cell sequencing reveals a transitional profibrotic macrophage. *Nat. Immunol.* **20**, 163–172 (2019).

8. G. Monaco, *et al.*, RNA-Seq Signatures Normalized by mRNA Abundance Allow Absolute Deconvolution of Human Immune Cell Types. *Cell Rep.* **26**, 1627-1640.e7 (2019).

9. F. Krueger, TrimGalore.

10. R. Patro, G. Duggal, M. I. Love, R. A. Irizarry, C. Kingsford, Salmon: fast and bias-aware quantification of transcript expression using dual-phase inference. *Nat. Methods* **14**, 417–419 (2017).

11. C. Soneson, M. I. Love, M. D. Robinson, Differential analyses for RNA-seq: transcript-level estimates improve gene-level inferences. *F1000Research* **4**, 1521 (2015).

12. S. Andrews, Fastqc.

13. P. Ewels, M. Magnusson, S. Lundin, M. Käller, MultiQC: summarize analysis results for multiple tools and samples in a single report. *Bioinforma. Oxf. Engl.* **32**, 3047–3048 (2016).

14. I. Lindeman, *et al.*, BraCeR: B-cell-receptor reconstruction and clonality inference from single-cell RNA-seq. *Nat. Methods* **15**, 563–565 (2018).

15. M. J. T. Stubbington, *et al.*, T cell fate and clonality inference from single-cell transcriptomes. *Nat. Methods* **13**, 329–332 (2016).

16. A. Dobin, *et al.*, STAR: ultrafast universal RNA-seq aligner. *Bioinforma. Oxf. Engl.* **29**, 15–21 (2013).

17. G. La Manno, *et al.*, RNA velocity of single cells. *Nature* **560**, 494–498 (2018).

18. V. Bergen, M. Lange, S. Peidli, F. A. Wolf, F. J. Theis, Generalizing RNA velocity to transient cell states through dynamical modeling. *Nat. Biotechnol.* **38**, 1408–1414 (2020).

19. R. A. Amezquita, *et al.*, Orchestrating single-cell analysis with Bioconductor. *Nat. Methods* **17**, 137–145 (2020).

20. J. Rainer, L. Gatto, C. X. Weichenberger, ensembldb: an R package to create and use Ensembl-based annotation resources. *Bioinformatics* **35**, 3151–3153 (2019).

21. A. T. L. Lun, D. J. McCarthy, J. C. Marioni, A step-by-step workflow for low-level analysis of single-cell RNA-seq data with Bioconductor (2016) https:/doi.org/10.12688/f1000research.9501.2 (May 23, 2023).

22. D. J. McCarthy, K. R. Campbell, A. T. L. Lun, Q. F. Wills, Scater: pre-processing, quality control, normalization and visualization of single-cell RNA-seq data in R. *Bioinformatics* **33**, 1179–1186 (2017).

23. A. Kassambara, *et al.*, RNA-sequencing data-driven dissection of human plasma cell differentiation reveals new potential transcription regulators. *Leukemia* **35**, 1451–1462 (2021).

24. N. T. Gupta, *et al.*, Change-O: a toolkit for analyzing large-scale B cell immunoglobulin repertoire sequencing data. *Bioinformatics* **31**, 3356–3358 (2015).

25. , Phylogenetic analysis of migration, differentiation, and class switching in B cells | PLOS Computational Biology (May 23, 2023).

26. K. B. Hoehn, *et al.*, Repertoire-wide phylogenetic models of B cell molecular evolution reveal evolutionary signatures of aging and vaccination. *Proc. Natl. Acad. Sci.* **116**, 22664–22672 (2019).

27. D. Merkel, Docker: lightweight Linux containers for consistent development and deployment. *Linux J.* **2014**, 2:2 (2014).

28. T. H. Olsen, F. Boyles, C. M. Deane, Observed Antibody Space: A diverse database of cleaned, annotated, and translated unpaired and paired antibody sequences. *Protein Sci. Publ. Protein Soc.* **31**, 141–146 (2022).

29. T. H. Olsen, B. Abanades, I. H. Moal, C. M. Deane, KA-Search: Rapid and exhaustive sequence identity search of known antibodies. 2022.11.01.513855 (2022).

30. M.-P. Lefranc, *et al.*, IMGT unique numbering for immunoglobulin and T cell receptor variable domains and Ig superfamily V-like domains. *Dev. Comp. Immunol.* **27**, 55–77 (2003).

31. K. Fischer, *et al.*, Microfluidics-enabled fluorescence-activated cell sorting of single pathogen-specific antibody secreting cells for the rapid discovery of monoclonal antibodies. 2023.01.10.523494 (2023).

32. , Clonally expanded CD8 T cells patrol the cerebrospinal fluid in Alzheimer's disease | Nature (May 23, 2023).

33. H. Wickham, ggplot2: Elegant Graphics for Data Analysis (2009).

34. T. Lin Pedersen, ggforce: Accelerating "ggplot2" (2022).

35. F. Briatte, ggnetwork: Geometries to Plot Networks with "ggplot2" (2023).

36. A. Theorell, Y. T. Bryceson, J. Theorell, Determination of essential phenotypic elements of clusters in high-dimensional entities-DEPECHE. *PloS One* **14**, e0203247 (2019).

37. R. Kolde, Pheatmap: pretty heatmaps. *R Package Version* **1**, 726 (2012).

38. T. J. Aragon, epitools: Epidemiology Tools (2020).
